# Reducing the Carbon Footprint of Academic Conferences: The Example of the American Society of Tropical Medicine and Hygiene

**DOI:** 10.4269/ajtmh.20-1013

**Published:** 2020-10-09

**Authors:** Teun Bousema, Prashanth Selvaraj, Abdoulaye A. Djimde, Derya Yakar, Brittany Hagedorn, Abigail Pratt, Didier Barret, Kate Whitfield, Justin M. Cohen

**Affiliations:** 1 Department of Microbiology, Radboud University Medical Centre, Nijmegen, The Netherlands;; 2 Institute for Disease Modeling, Bellevue, Washington;; 3 University of Science, Techniques and Technology of Bamako, Bamako, Mali;; 4 Department of Radiology, University Medical Center Groningen, Groningen, The Netherlands;; 5 Bill & Melinda Gates Foundation, Seattle, Washington;; 6 Institut de Recherche en Astrophysique et Planétologie, Centre National de la Recherche Scientifique, Toulouse, France;; 7 ISGlobal, Hospital Clínic - Universitat de Barcelona, Barcelona, Spain;; 8 Clinton Health Access Initiative, Boston, Massachusetts

## Abstract

We calculated carbon emissions associated with air travel of 4,834 participants at the 2019 annual conference of the American Society of Tropical Medicine and Hygiene (ASTMH). Together, participants traveled a total of 27.7 million miles or 44.6 million kilometers. This equates to 58 return trips to the moon. Estimated carbon dioxide equivalent (CO_2_e) emissions were 8,646 metric tons or the total weekly carbon footprint of approximately 9,366 average American households. These emissions contribute to climate change and thus may exacerbate many of the global diseases that conference attendees seek to combat. Options to reduce conference travel–associated emissions include 1) alternating in-person and online conferences, 2) offering a hybrid in-person/online conference, and 3) decentralizing the conference with multiple conference venues. Decentralized ASTMH conferences may allow for up to 64% reduction in travel distance and 58% reduction in CO_2_e emissions. Given the urgency of the climate crisis and the clear association between global warming and global health, ways to reduce carbon emissions should be considered.

## INTRODUCTION

Climate change has a direct negative impact on global health.^1,2^ The temperature of the planet is rising, and the pace of climate change is accelerating.^3–8^ There has been an increase in atmospheric carbon dioxide (CO_2_) of nearly 50% since 1990, and the last decade was the warmest decade ever recorded.^9^ Higher temperatures are associated with lower agricultural crop yields, affecting nutrition, food, and water security^3^ and may also worsen growth faltering in malnourished children.^10^ In some settings, increasing temperatures may also increase vector-borne diseases including malaria, dengue, and chikungunya.^1,11,12^ The negative consequences of climate change are not equally distributed across the globe. In recent years, sub-Saharan Africa has experienced more frequent and more intense climate extremes than in prior decades.^8^ Thousands of lives and disability-adjusted life years have already been lost because of the consequences of climate change in Africa.^4,12^

Air travel generates significant greenhouse gas emissions and thereby exacerbates the problems the global health community is aiming to solve. Many academics and public health practitioners are part of a hypermobile lifestyle that involves frequent air travel. Although some travel is essential for global health research, travel restrictions imposed by the COVID-19 pandemic forced communities to consider alternative modes of connecting with colleagues and partners. The annual meeting of the American Society of Tropical Medicine and Hygiene (ASTMH) is the largest conference in global health and plays an important role in bringing together international experts and students to share the latest advances.

## TRAVEL-ASSOCIATED CARBON FOOTPRINT OF THE ASTMH 2019

**Figure 1.**
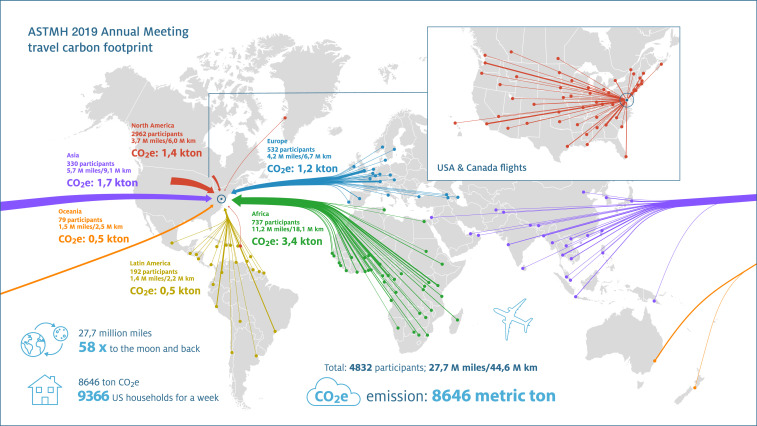
Travel to the American Society of Tropical Medicine and Hygiene 2019 conference. Data from participants and their country, state, or province of origin were used to estimate their travel distance and the travel-associated CO_2_e emissions. Each line connects a single airport in the country, state, or province of departure with the conference venue in Washington, DC. The thickness of the line reflects the number of participants; the travel route is stylized, although actual travel distance based on available flights (including flight legs) was used for calculations. CO_2_e = carbon dioxide equivalent.

We calculated air travel distances and their associated carbon emissions for the 68th annual meeting of the ASTMH that was held from November 20 to 24, 2019 in National Harbor, Maryland. For all participants, information on the country (international participants), state (U.S. participants), or province (Canadian participants) of origin formed the starting point to select the most likely airport of departure, defined as the international airport in the country/state/province with the largest average number of flights annually. We then selected the flight option with fewest changes using an online travel planner^13^; for some long-haul flights, this is likely to generate a conservative estimate of travel distance and carbon emissions because journeys with multiple connections are often cheaper.^14^ For U.S. conference participants from Delaware, District of Columbia, Maryland, and Virginia, we conservatively assumed no air travel. We then calculated distances between the presumed airport of departure and Washington Dulles International Airport along a geodesic (great circle) path.^15^ The distance of each flight leg was translated to CO_2_ equivalent (CO_2_e) emissions using the mean emission factors per kilometer and per passenger of three independent sources: the U.K. Department for Environment, Food and Rural Affairs, the French Agence de la transition écologique, and the nonprofit organization myclimate. These emission factors take into account 1) direct emission of radiatively active substances (e.g., CO_2_), 2) emission of chemical species that alter radiatively active substances, and 3) emission of substances that trigger generation of aerosol particles or change natural clouds.^14^ We conservatively assumed economy travel only; premium economy, business, and first-class seating are associated with ∼1.5-, ∼2-, and ∼3-fold higher emissions, respectively.^14^ The 4,834 conference participants originated from 110 countries across six continents (Figure 1). Their combined return flights accounted for 27.7 million miles or 44.6 million kilometers flown. This distance equals 58 return trips to the moon (assuming an average distance of 384,400 km or 238,855 miles^16^). Total CO_2_e emissions from this air travel were estimated at 8,646 metric tons, equaling the total weekly carbon footprint of approximately 9,366 average American households (assuming an annual CO_2_ emission of 48t CO_2_/household/year).^17^

## ALTERNATIVES TO AN ANNUAL CENTRAL CONFERENCE

The COVID-19 pandemic has forced a halt to international travel and necessitated a switch to virtual meetings. For 2020, the ASTMH meeting will be held virtually. The experience will help us consider the trade-offs between virtual and in-person meetings and offer an opportunity to develop a sustainable model to implement beyond the COVID-19 pandemic. Several options may be considered to reduce the carbon footprint associated with future conferences.^18^ Although a complete replacement of in-person meetings with virtual meetings can be considered, we are of the opinion that some in-person contact is needed for networking and productive scientific discourse. We thus consider three alternatives to the way ASTMH meetings have been organized in the past: 1) alternating in-person and online conferences, 2) offering a hybrid in-person/online conference, and 3) decentralizing the conference with multiple conference venues. Alternating in-person and online conferences would allow for the annual exchange of scientific progress and new insights with a biennial opportunity to meet in person. This option would immediately cut carbon emissions by half and has an example in the biannual European Congress on Tropical Medicine and International Health that targets a highly similar audience. A second option is organizing an annual in-person meeting while concurrently offering an online option. This would provide participants with maximum flexibility to choose their conference format and has examples in Keystone Symposia that routinely make part of their content available online. Importantly, the virtual option may increase conference accessibility and attract participants who would otherwise not attend the meeting, illustrated by the > 4-fold increase in participants experienced by the American Physical Society when organizing their 2020 conference online.^19^ A third option would be a decentralized conference. Decentralized conferences would allow for in-person networking in smaller groups with international colleagues from the same region and can sustain the quality of a central meeting if keynote speakers are allocated to different venues and video content from all venues is available. It would be important to ensure that participants of conferences that are hosted in low-resource settings have access to the same breadth of presentations as those attending conferences elsewhere.

**Figure 2.**
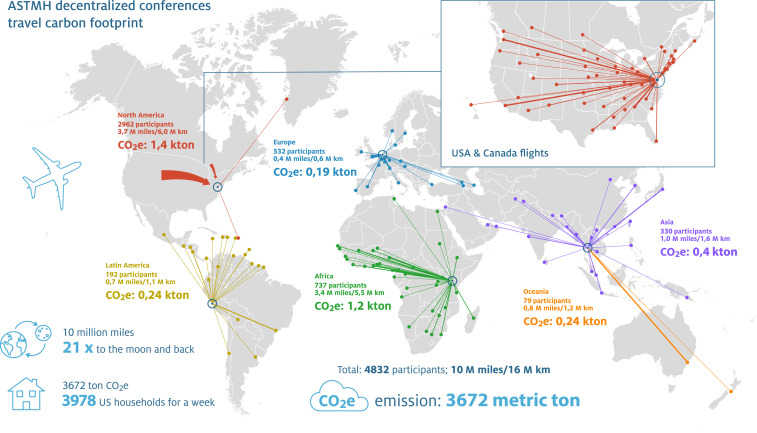
Estimated impact of decentralized American Society of Tropical Medicine and Hygiene conferences. Data from participants and their country, state, or province of origin were used to explore the impact on total travel distance and CO_2_e emissions if the conference was held at multiple venues in Washington, DC, Lima, Amsterdam, Nairobi, and Bangkok. The thickness of the line reflects the number of participants; the travel route is stylized, although actual travel distance based on available flights (including flight legs) was used for calculations. CO_2_e = carbon dioxide equivalent.

To explore the potential impact of this third option, we recalculated the travel distance and carbon emissions (Figure 2) assuming conference venues in Washington, DC (for participants from North America), Lima, Peru (for participants from Latin America), Bangkok, Thailand (for participants from Asia and Oceania), Nairobi, Kenya (for participants from Africa), and Amsterdam, the Netherlands (for participants from Europe). We used the approach described previously and assumed air travel for all international travel, even when train travel options exist, for example, in Europe. We estimated that a decentralized option could reduce travel distances by 64% (from 27.7 million miles for the centralized option to 10.0 million miles for the decentralized option) and would reduce carbon emissions by approximately 58% (from 8,646 metric tons CO_2_e for the centralized option to 3,672 metric tons for the decentralized option). Compared with the 2019 ASTMH conference in Maryland, decentralized conference venues would result in a substantial reduction in the mean distance traveled for participants coming from all regions outside the United States (Africa: reduction from 7,631 to 2,317 miles for a single journey, Asia: 8,580 to 1,505 miles, Europe: 3,923 to 342 miles, Oceania (9,743 to 4,858 miles), and Latin America (3,522 to 1,765 miles).

## CONCLUSION

International conferences play an important role in enabling sharing of research findings, fostering collaborations, and building working relationships. Travel to the 2019 ASTMH conference equated to 58 trips to the moon and back. Participants from Africa, in particular, who often lack direct flight options, had to invest disproportionately in travel time and expenses. At the same time, we consider the presence of participants from all continents—especially those from disease-endemic countries—an essential feature of the ASTMH meeting, which focuses on tropical medicine. The carbon cost associated with travel is considerable for a conference that focuses on global health, which is directly and indirectly affected by climate change. The COVID-19 pandemic has accelerated the use of digital conferencing options to replace or complement in-person meetings, and the conferencing technology will continue to improve. The immediate climate crisis demands that we identify sustainable alternatives to large in-person conferences now, taking advantage of the learning opportunity that the pandemic provides us. Options to reduce the post-pandemic carbon footprint of large conferences like the ASTMH could take the form of hybrid conferences that maximize the advantages of both in-person and digital conferencing. Alternating in-person and digital conferences or offering both options concurrently are viable approaches. Both approaches facilitate frequent scientific interactions while reducing travel and at the same time improving conference accessibility.^19^ Last, a decentralized option may offer advantages. Although there will be considerable logistic challenges in organizing multiple conferences and caution is required to ensure participants from all continents have equal benefits,^18^ the gains in terms of travel costs, emissions, and time are considerable. This decentralized option may be alternated with a central conference. In conclusion, although in-person meetings will remain vital for dissemination of knowledge and productive research collaborations, the ecological impact of conference travel is considerable and demands action. The global health community can lead the way in innovating and establishing a sustainable model to advance the global health agenda without exacerbating the global climate crisis.
